# Long-term parental satisfaction with adenotonsillectomy: a population study

**DOI:** 10.1007/s11325-015-1149-3

**Published:** 2015-03-06

**Authors:** Wojciech Kukwa, Andrzej Kukwa, Adam Galazka, Anna M. Czarnecka, Antoni Krzeski, Ewa Migacz, Stacey L. Ishman

**Affiliations:** 1 Department of Otorhinolaryngology, Medical University of Warsaw, 19/25 Stepinska Street, 00-739 Warsaw, Poland; 2 Department of Otolaryngology and Head and Neck Disease, School of Medicine, University of Varmia and Mazuria, 30 Warszawska Street, 10-082 Olsztyn, Poland; 3 Laboratory of Molecular Oncology, Department of Oncology, Military Institute of Medicine, 128 Szaserow Street, 04-141 Warsaw, Poland; 4 Healthy Sleep Foundation, 10/55 Dworkowa Street, 05-077 Warsaw, Poland; 5 Division of Pediatric Otolaryngology - Head & Neck Surgery and Pulmonary Medicine, Cincinnati Children’s Hospital Medical Center, Cincinnati, OH USA; 6 Department of Otolaryngology – Head & Neck Surgery, University of Cincinnati College of Medicine, Cincinnati, OH USA; 7 Medical University of Warsaw, 10/55 Dworkowa Street, 05-077 Warsaw, Poland

**Keywords:** Sleep-disordered breathing, Snoring, Children, Adenotonsillectomy, Questionnaire

## Abstract

**Objective:**

This study reports the presence of sleep-disordered breathing (SDB) symptoms among first graders. We evaluated the severity of symptoms and parental satisfaction in children who had undergone adenotonsillectomy (T&A) compared to children who never had T&A.

**Methods:**

A population-based, cross-sectional study was conducted. Parent-reported questionnaire data including age, weight, height, BMI, history of T&A, and SDB symptoms (snoring intensity, observed dyspneas, mouth breathing during sleep) were analyzed.

**Results:**

Of 3580 eligible children, 2504 (69.9 %) returned questionnaires. Two hundred fifty-nine (10.3 %) children had a history of T&A. Within this group, 76 % of parents were still satisfied with their child’s outcome after surgery. The satisfaction rate decreased from 88.9 % in those who had surgery within 1 year to 71.3 % in those who had surgery ≥3 years prior. The mean values of intensity for all analyzed SDB symptoms on a five-point scale were significantly higher for children who had ever undergone T&A when compared to those who never underwent surgery [e.g., snoring (2.11 vs 1.87; *P* = 0.0004), dyspneas (1.64 vs 1.22; *P* < 0.0001), and mouth breathing during sleep (2.95 vs 2.58; *P* < 0.0001)]. For those who had undergone T&A, 24 % of caregivers were not satisfied with the surgical outcome and the symptom intensity was highest (snoring = 3.16, dyspneas = 2.20, and mouth breathing = 4.23) for these children.

**Conclusions:**

The mean SDB symptom intensity was significantly higher in children who had undergone T&A when compared to those who had not. The elevated symptom intensity in those children whose caregivers were not satisfied after T&A suggests possible SDB recurrence and need for further evaluation.

## Introduction

Snoring is a primary symptom of sleep-disordered breathing (SDB) in children. SDB comprises a spectrum of nocturnal ventilation disorders, which range from primary snoring (PS) and upper airway resistance syndrome (UARS) to obstructive sleep apnea (OSA) [1]. SDB disorders have been shown to have a negative influence on the growth and cognitive development in children. Typical and well-described sequelae of pediatric SDB include reduced neuropsychological and cognitive function, negative cardiovascular effects, and growth deficits [2]. Adenotonsillectomy (T&A) is considered as the first-line method of treatment for all forms of SDB [3, 4].

In the USA, approximately 530,000 adenotonsillectomies are performed each year. Of these, 77 % are performed for breathing disturbances during sleep [5, 6]. Even though most of the published data show significant improvements in snoring and apnea, as well as neurocognitive function and cardiovascular complications, residual upper airway obstruction with persistent SDB is quite common. The likelihood of cure after T&A in children with OSA is decreased for those who are overweight and obese, those who are 7 years of age or older at the time of surgery, and those with high preoperative apnea-hypopnea index (AHI) [2, 7–9]. Most of the studies measuring the effect of T&A on polysomnographic (PSG) or clinical symptoms in children were performed soon after the surgery. However, few reports showing the long-term effects of surgery on SDB severity and upper airway symptoms have been published.

The aim of this study was to evaluate the severity of SDB symptoms, including snoring, observed dyspneas, and mouth breathing during sleep in children who had undergone T&A, and to compare them to a group who never had T&A. Additionally, we evaluated the differences in symptom severity compared to parents’ long-term satisfaction with surgery for children who had previously undergone T&A.

## Methods

### Participants

Questionnaires were distributed to the parents of every child enrolled in the first grade in 34 primary schools located in three different cities in Poland with similar economic status. School authorities distributed the questionnaires to the parents along with a brochure educating them about pediatric SDB. The questionnaire included the parent’s written informed consent. These were returned and analyzed. The data were collected between May 2011 and January 2014. Because >95 % of the population of Poland is Caucasian, we did not collect information on race or ethnicity. The study was approved by the bioethical committee, local institutional review board, and the regional director of education.

### Procedure

This study was designed as a population-based cross-sectional study. After an informational campaign, the parents of the recruited first graders were asked to fill out a questionnaire regarding demographics, symptoms during sleep, and history of T&A. The questionnaire included a statement regarding the voluntary nature of the questionnaire, and the parents were asked to complete a separate written informed consent. Parents were also asked to provide the most recent age, height, and weight of their child and to report the presence of snoring. They were then asked to grade snoring intensity, mouth breathing during sleep, and observed dyspnea during sleep on a five-point scale (1, never; 2, occasionally; 3, sometimes; 4, often; and 5, very often/always). No additional instruction was given on the descriptors in the questionnaire. Habitual snoring was defined as responses of 4 (often) or 5 (very often/always) on this scale. All data were stored in a protected computer database, and the data were checked for errors by a trained technician.

For those parents whose children had undergone previous T&A, we asked for the duration of time since the surgery and about short- and long-term nocturnal breathing and SDB outcomes since surgery. The duration of time since surgery was categorized as 3 years ago or longer, 2 to less than 3 years ago, 1 to less than 2 years ago, or less than 1 year.

### Statistical analysis

Statistical analysis was performed using Statistica 8.0 software (StatSoft, Inc.). The variables were tested for significant differences using Kruskal–Wallis one-way analysis of variance or the Mann–Whitney test. *P* values of <0.05 were considered to indicate statistical significance.

## Results

Three thousand five hundred eighty questionnaires were distributed, and 2504 (69.9 %) were completed and successfully returned. The mean age for the children was 7.12 ± 0.65 years. The mean height was 128.55 ± 7.02 cm, and the mean weight was 26.64 ± 5.59 kg. The mean BMI was thus 16.04 ± 2.44. Habitual snoring was reported in 158 (6.31 %) of the children.

A history of T&A was reported in 259 (10.3 %) children. Of these children, 112 (43 %) had the surgery more than 3 years before the survey and only 27 (11 %) had the surgery during the year immediately prior to completion of the survey (Table 1). The percentage of parents who were still satisfied with the surgery is shown in Table 1.

**Table 1:** Duration of time since surgery for those children who underwent previous T&A and rate of satisfaction for caregivers in each group

	Duration of time since surgery			
	>3 years	2–3 years	1–2 years	<1 year
Children with previous T&A (*N*, %)	112 (43)	73 (28)	47 (18)	27 (11)
Satisfaction after surgery (*N*, %)	80 (71.3)	53 (72.6)	36 (76.6)	24 (88.9)

*T&A* adenotonsillectomy

The mean intensity of all analyzed SDB symptoms, including snoring, observed dyspneas, and mouth breathing during sleep, was significantly higher for those children who underwent previous T&A when compared to children who never had T&A (Table 2). Further stratification of children who underwent previous T&A into those who improved following surgery and those who did not improve (or did not have sustained improvement) can be found in Table 3. We found that the mean values of SDB symptoms in those who were symptomatic at the time of evaluation were considerably higher than those in children whose parents were satisfied with the results of T&A and those who never underwent previous T&A (Table 3). Moreover, there was no significant difference in BMI between these three groups.

**Table 2:** Mean values for body mass index (BMI), snoring intensity, witnessed dyspneas, and mouth breathing during sleep in children who had previous adenotonsillectomy (T&A) when compared to those who did not have previous T&A

	Children with previous T&A	Children without previous T&A	*P* value
N	259	2245	
BMI	16.39	15.98	0.0699
Snoring intensity	2.11	1.87	0.0004
Witnessed dyspneas	1.64	1.22	<0.0001
Mouth breathing	2.95	2.58	<0.0001

*T&A* adenotonsillectomy, *BMI* body mass index

**Table 3:** Mean values for body mass index (BMI), snoring intensity, witnessed dyspneas, and mouth breathing during sleep in the three groups of children: (1) children who underwent previous T&A and whose caregivers are still satisfied with the results of the surgery, (2) children who underwent previous T&A and whose caregivers are not satisfied with the results of surgery, and (3) children who did not undergo previous T&A

	Asymptomatic children with previous T&A	Symptomatic children with previous T&A	Children who never underwent T&A	*P* value
N	193	66	2245	
BMI	16.30	16.72	15.99	0.1720
Snoring intensity	1.79	3.16	1.87	<0.0001
Witnessed dyspneas	1.48	2.20	1.22	<0.0001
Mouth breathing	2.57	4.23	2.58	<0.0001

*T&A* adenotonsillectomy, *BMI* body mass index

## Discussion

Adenotonsillectomy remains to be one of the most commonly performed surgeries in children. In the present study, 10.3 % of children in the first grade had undergone previous T&A. The lifetime likelihood of undergoing T&A varies between countries [10] from a low of approximately 2 % in Canada to 12 % in Northern Ireland and as high as 15 % in Finland [11]. In the USA, where just over four million children are born each year, 530,000 tonsillectomies and 130,000 adenoidectomies are performed [12]; more than 75 % of these are performed for upper airway obstruction indications [6]. In addition, most of the children in our study underwent T&A at the age of 4 or younger.

We also measured parental satisfaction with T&A and found that 76 % of all parents were satisfied that SDB symptoms have adequately resolved. This supports previous studies, which demonstrate that adenotonsillectomy is a highly effective treatment for SDB symptoms in children and is frequently the only treatment required for SDB. Meta-analyses of PSG findings after T&A report that short-term success rates are 66.3 to 82.9 % [13, 14]. Although complete resolution is not achieved for a significant number of children, surgery results in significant improvements in AHI and associated clinical symptoms of SDB even for those without complete resolution. When questionnaires are used to measure subjective improvements in quality of life after T&A, the parental satisfaction levels are even higher [15]. Wolfsberger et al. reported a very high rate of parent satisfaction after adenotonsillectomy (91 %) whether surgery was performed for recurrent tonsillitis or obstructive sleep apnea [16].

The number of parents satisfied with the long-term outcome of the surgery varied depending on the time since surgery was completed. In the first year after surgery, 89 % of parents were happy with the resolution of symptoms. This perception of subjective success decreased over time but remained high at 71 % for children who underwent surgery three or more years prior to evaluation. A 2008 study by Amin et al. [17] prospectively followed a group of children with SDB after T&A and found an increasing number of recurrences during the first year after surgery. Similarly, adolescents who were successfully treated with surgical or orthodontic therapy during childhood have been shown to have recurrence of symptoms over time [18].

We found that all of the studied symptoms of SDB were significantly higher in the group of children who had undergone T&A in the past. These results are similar to those of Liukkonen et al. [11] who found that snoring children were significantly more likely to have undergone previous adenoidectomy than non-snoring children.

A closer look at the children who were reported to be symptomatic after T&A found that 24 % of their parents were not satisfied with the long-term outcomes of T&A. These children had higher levels of all measured SDB symptoms when compared to children who were asymptomatic after T&A and those who had never undergone TA&. The mean score for mouth breathing in this subgroup was almost twice as high as in the other two groups (4.23 vs 2.57 and 2.58, respectively). The scores for symptomatic children suggest that they frequently had symptoms: often (equivalent to a score of 4) or very often/always (equivalent to a score of 5). Similarly elevated mean values were observed for snoring and observed dyspneas for symptomatic children after T&A when compared to the other two groups. This suggests that long-term follow-up is important for children after adenotonsillectomy as the surgery may not completely resolve the symptoms of SDB and that symptomatic relief decreases over time. According to the literature, persistent or recurrent symptoms are often attributable to nasal pathology [15, 19], but no population studies were performed to prove this observation.

Further evaluation of factors which might affect the relationship between persistence or recurrent of SDB symptoms after T&A led us to look at BMI. Increased BMI is one of the most important risk factors for the persistence or recurrence of breathing disorders during sleep in children undergoing adenotonsillectomy. Previous studies have found that preoperative obesity is the most significant risk factor for postoperative residual sleep apnea for children [7, 20, 21]. Only one study by Apostolidou et al. reported that obesity did not predict an increased likelihood of persistent OSA after T&A in children [22]. In our study, BMI was not a risk factor for the recurrence of SDB symptoms as there was no significant difference in BMI between any of the three groups of children.

Limitations of the study include the fact that SDB severity was measured solely on the basis of a questionnaire, and sleep studies were not used to evaluate these children. However, it would not have been possible to get sleep studies on all these children and this method is similar to that used in other population-based studies in pediatric sleep medicine [11, 16]. We do recognize that parental satisfaction may be affected by many factors, but we would expect many of these confounders to be nondifferential between responders. In addition, there are no available validated questionnaires in Polish to evaluate SDB; therefore, we modeled the current survey on the pediatric sleep questionnaire (PSQ) and the OSA-18. Unfortunately, given limited resources, it was not realistic for us to validate this survey in this large population. Moreover, children who had a history of T&A underwent surgery in many different ENT departments. Therefore, different surgical approaches may have been used to treat them. Since it has been shown that there can be different outcomes after surgery depending on the type of T&A surgery (e.g., total tonsillectomy, tonsillotomy, tonsillectomy with pillar closure) performed [4, 23], this may have influenced the degree of SDB resolution after T&A.

Despite these limitations, the present study highlights the fact that children, especially those who had a history of T&A, should be closely evaluated for persistent or recurrent symptoms of SDB. In addition, these children may benefit from long-term follow-up, especially for those who have symptom in the first year after T&A.

## Conclusions

In the present study, the rate of T&A in Polish children was high at 10.3 %. Most of the children benefited from surgery and remained asymptomatic for at least 3 years after T&A. However, for those children who had undergone previous T&A, we found that 24 % of parents were dissatisfied with the long-term outcome of T&A. This general dissatisfaction correlated with significantly higher SDB symptom intensity. These findings suggest that further study is needed to examine symptomatic children and that long-term follow-up for SDB may be warranted in children after T&A. In addition, the creation of an evidence-based protocol to diagnose and treat these children after T&A would be useful.
